# Replicability and reproducibility of predictive models for diagnosis of depression among young adults using Electronic Health Records

**DOI:** 10.1186/s41512-023-00160-2

**Published:** 2023-12-05

**Authors:** David Nickson, Henrik Singmann, Caroline Meyer, Carla Toro, Lukasz Walasek

**Affiliations:** 1 WMG, University of Warwick, Coventry, UK; 2 Department of Experimental Psychology, University College London, London, UK; 3 Warwick Medical School, University of Warwick, Coventry, UK; 4 Department of Psychology, University of Warwick, Coventry, UK

**Keywords:** Depression, Electronic health records, Machine learning, Predictive modelling, Replicability, Reproducibility

## Abstract

**Background:**

Recent advances in machine learning combined with the growing availability of digitized health records offer new opportunities for improving early diagnosis of depression. An emerging body of research shows that Electronic Health Records can be used to accurately predict cases of depression on the basis of individual’s primary care records. The successes of these studies are undeniable, but there is a growing concern that their results may not be replicable, which could cast doubt on their clinical usefulness.

**Methods:**

To address this issue in the present paper, we set out to reproduce and replicate the work by Nichols et al. (2018), who trained predictive models of depression among young adults using Electronic Healthcare Records. Our contribution consists of three parts. First, we attempt to replicate the methodology used by the original authors, acquiring a more up-to-date set of primary health care records to the same specification and reproducing their data processing and analysis. Second, we test models presented in the original paper on our own data, thus providing out-of-sample prediction of the predictive models. Third, we extend past work by considering several novel machine-learning approaches in an attempt to improve the predictive accuracy achieved in the original work.

**Results:**

In summary, our results demonstrate that the work of Nichols et al. is largely reproducible and replicable. This was the case both for the replication of the original model and the out-of-sample replication applying NRCBM coefficients to our new EHRs data. Although alternative predictive models did not improve model performance over standard logistic regression, our results indicate that stepwise variable selection is not stable even in the case of large data sets.

**Conclusion:**

We discuss the challenges associated with the research on mental health and Electronic Health Records, including the need to produce interpretable and robust models. We demonstrated some potential issues associated with the reliance on EHRs, including changes in the regulations and guidelines (such as the QOF guidelines in the UK) and reliance on visits to GP as a predictor of specific disorders.

## Background

With a lifetime prevalence of 20% across all ages, depressive disorders are now among the most common mental health conditions [1–3]. The burden caused by depression is considerable, both in terms of DALYs (Disability-adjusted life years) and YLDs (Years of healthy life lost due to disability), [4, 5]. Beyond its significant personal and social impact, depression carries substantial economic costs. In 2007, the total cost of depression in England was £7.5 billion. Of this, £1.7 billion was spent on health services and £5.8 billion was lost due to the ensuing reduction in economic output [6, 7].

Of particular concern are the growing rates of depression among adolescents and young adults. Some prevalence estimates include 4% in Spain [8, 9], 6% in the UK [10], and 10% in Australia [11]. More recent estimates (based on US data from 2020) show that 17.2% of 18–25-year-olds, and 16.9% of 12–17-year-olds, experienced depression [12]. Latest estimates from the UK show that nearly a third (31%) of 16–24-year-old females suffered from depression between 2017 and 2018 [13]. These rates are alarming, in large part because those who experience depression (or its precursors) in early life are more likely to suffer from it at a later age [14–17]. Despite these trends, interventions targeting depression among young people are uncommon, with most of the existing efforts instead focusing on prevention [18].

An obvious challenge for early intervention is how to diagnose depression early in one’s lifetime. In the case of child and adolescent mental health in the UK, primary practitioners often lack the necessary training, knowledge, and confidence to diagnose depressive disorders [19]. It has also been shown that more young people look for mental health support now than in the past, increasing pressure on diagnostic and treatment resources either because of a genuine increase in incidence or because of lowered barriers to seeking help [20]. In the UK, a further barrier to early diagnoses of depression is the general lack of funding and prioritization of other health issues. Consequently, it is estimated that merely a third of adolescent depression is actually identified [21].

A new promise for streamlining and improving early diagnoses, especially among young people, involves the use of Electronic Health Records (EHRs). EHRs are digital databases of systematically coded patient records, often supplemented with free text comments made by practitioners. Since the 2000s, EHRs have become widely adopted (> 90%) in the USA, UK, Netherlands, Australia, and New Zealand [22]. Although EHRs differ between jurisdictions, they typically include individual-level historical data about patient’s health conditions, results of medical tests, records of treatments and special care, details about a person’s lifestyle (e.g., smoking, drinking), and individual demographic characteristics (e.g., age, address). In more recent years, EHRs have been used by researchers to train classification models for predicting diagnoses of various disorders, including cardiovascular conditions [23, 24] diabetes [25], dementia [26], first episode of psychosis [27, 28], depression [29], and childhood mental health problems [30]. Overall, these efforts show a lot of promise by offering a predictive/diagnostic performance that is comparable to or better than that typically achieved in non-specialist primary care [31–33]. With the growing ubiquity of EHRs, nearing 100% in Western countries (22), combined with the recent advances in machine learning (ML) methods, one can expect that this approach will continue to improve early diagnosis for mental health.

One potential but significant barrier to realizing the potential of EHR-driven predictive modeling is whether methodologies used to train large models on big health data are reproducible and whether the results of such efforts are replicable. Despite the growing concern about the reliability of many findings from experimental psychology [34, 35], clinical psychology [36], genomics [37], and digital medical sciences [38–40], we are not aware of a single attempt to replicate research that trains predictive models of depression on the basis of EHRs. Beyond the obvious scientific and applied value of ascertaining the conclusions of existing studies [41, 42] the reliance on EHRs and ML methods poses a unique risk to the validity and reliability of the previous research. First, various aspects of EHR data can change over time, with consequences for the predictive value of the existing models. For example, EHR data can vary over time due to changes in prescribing and diagnostic guidance, due to external shocks in the environment (e.g., pandemics, economic factors), or simply because of errors in data entry [43]. In fact, even changes in the design of EHR data entry systems could influence diagnostic code selection based on, for example, misclassification errors [44]. It is not difficult to see that such factors could have a significant impact on the predictive accuracy of models trained on a single vertical slice of the EHRs. Second, reliance on big healthcare data and ML methods introduces many degrees of freedom for the researchers (for a review, see Gundersen and Kjensmo [45]. Indeed, insufficient information about the model fitting procedure, lack of transparency about predictor/feature selection, ambiguous data pre-processing steps, or lack of easily available and annotated code, are among many reasons why most of the existing ML applications are not reproducible [46, 47].

### Current research

In light of concerns about replicability and reproducibility of existing research, the goal of the present study is to replicate a study that combines ML and EHRs to predict depression among young adults (15–24 years). Our target study is that of Nichols et al. [48] [henceforth, NRCBM] who reported results of models trained on EHRs to predict depression among four groups of young people: females aged 15–18 (F 15–18), females aged 19–24 (F 19–24), males aged 15–18 (M 15–18), and males aged 19–24 (M 19–24). Unlike many existing replication attempts [49], our choice of the NRCBM was not motivated by the surprisingness of the original claims, or by any expectations (or concerns) about the validity of the conclusions drawn by the authors. Instead, we chose NRCBM for pragmatic reasons mostly, namely that we were able to gain access to a large sample from the same EHR database^1^

In their study, NRCBM acquired a fully anonymised matched case–control EHRs with details of patients aged between 15 and 24 from The Health Information Network database, THIN [50]. The authors were able to obtain data that covered the time interval between 1st of January 2000 and 31st of December 2012. In total, dataset included 67,321 cases and 192,135 controls, with further 31,241 cases and 89,113 controls used for model validation. The authors identified an initial set of 54 potential predictors of depression, based on the teams’ psychiatric and clinical experience of depression in young people and their knowledge of other findings from the relevant literature. The authors reduced their list of predictors based on their prevalence and by using a backward-stepwise logistic regression. NRCBM demonstrated promising results, reporting average AUC-ROC performance ranging from 0.699 and 0.719. The authors further reported a range of symptomatic and socio-economic factors predictive of depression that were common across all models, which included deprivation quintile, smoking status, depression-relevant symptoms (e.g., low mood, anxiety), somatic symptoms (e.g., headache, back pain), co-morbidities (e.g., diabetes, asthma), family and social factors (e.g., young carer, work stress), and other psychological conditions (e.g., OCD).

In the present study, we obtained a new (non-overlapping with the NRCBM dataset) of EHRs data from THIN, containing cases and matched controls for depression diagnosis among males and females aged between 15 and 24. By following NRCBM’s methodology, we pre-processed our data and fitted new regression models to the four subsets of the health records, stratified by age and gender. There are two outputs of this analysis. First, we report on our ability to reproduce each step of the analytical procedure described by NRCBM. We compare the list of final predictor variables following the pre-processing steps outlined by NRCBM, and we also assess the stability of the results from the backward-stepwise regression models. Second, we determine whether the main results reported by NRCBM are replicable. To this end, we present AUC-ROC curves from the newly fitted models, comparing these to the results reported by NRCBM. In addition, we also use coefficient estimates in the original study to make out-of-sample predictions on our own data and we report AUC-ROC curves based on this analysis.

Although backwards stepwise variable selection is a “traditional” [51] variable selection strategy, it is not without critics, e.g., [52–55]. Among the main complaints about stepwise procedures are that the selection of variables can be unstable, that stepwise procedure does not necessarily select the most important variables, and that stepwise procedures do not show the best prediction performance. Whereas the first of these issues can be addressed with a replication of NRCBM’s methodology, the latter two issues require the consideration of additional methods. Our secondary objective therefore is to extend the efforts of NRCBM to go beyond the stepwise logistic regression model and make comparisons with more advanced classification methods from the ML literature to predict depression. A range of different techniques were evaluated including LASSO (Least Absolute Shrinkage Selection Operator); Random Forest; Gradient Boosting; XGBoost; Rpart; and PRE (Prediction Rules Ensembles). All these models were assessed against the same criteria as the logistic regression models.

## Methods

Our methods section is structured as follows. We first provide an overview of the methods reported by NRCBM. We report on how the authors obtained and pre-processed the data prior to fitting their regression models. In the second part, we follow the same structure when discussing our own data and analysis.

In replicating this study, we have followed the guidelines given in the Transparent reporting of a multivariable prediction model (TRIPOD) for individual prognosis or diagnosis [56].

## NRCBM

### Data

NRCBM obtained their data from The Health Improvement Network (THIN), a database of anonymised primary care records in the UK. The obtained dataset comprised records gathered between 1st of January 2000 and 31st of December 2012, and included data from individuals between 15 and 24 years of age who were registered at a given practice for at least 1 year in that period. Data initially included records from 564 practices that were eligible by having at least 1 year’s worth of EHRs in the period specified above.

**Fig. 1:**
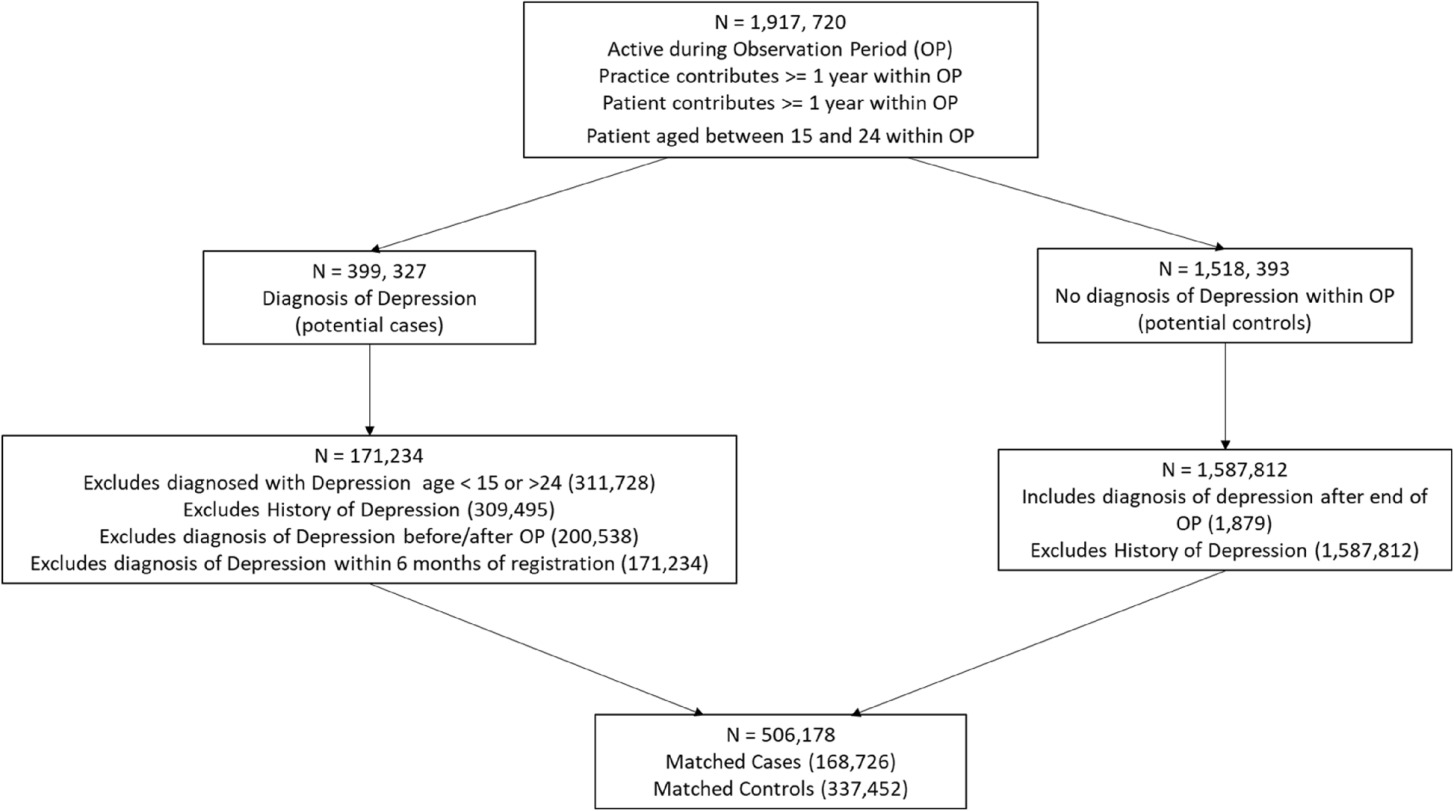
Cohort inclusion/exclusion criteria workflow – Replication Note: Observation period (OP) and extraction dates: Cohort observation start date 01–12-2008, Cohort observation end date 30–11-2020, Cohort start date for data extraction 01–01-1994, Cohort end date for data extraction 30–11-2020

The depression case was defined in terms of a combination of National Health Service (NHS) Read codes [57] and/or the prescription of an antidepressant (see NRCBM for details). In their data preparation stage, NRCBM excluded patients with diagnosis of psychosis, bipolar disorder, and hypomania. Furthermore, patients with a history of depression, who were diagnosed before the beginning of the study period (1st January 2000–21st of December 2012), and those who were diagnosed before the age of 15, were all excluded from the group of potential cases (see Fig. 1 in NRCBM for further details). After excluding patients on the basis of these criteria, the NRCBM data included 98,562 cases and 281,248 controls matched by practice, index date (i.e., the date of diagnosis of the case), gender, and age (up to ± 3 years). The final dataset had a 1:2.85 case to control ratio.

First NRCBM identified a set of “exposure variables” (or predictors) for depression. These were defined in terms of a combination of Read/drug codes and were grouped into the following categories: social deprivation, depression symptoms, somatic symptoms, co-morbidities, and family/social factors. The individual predictors are listed in Appendix 1.

NRCBM then identified the prevalence of the pre-selected predictor variables which were used in a two-step process. In the first step, the authors removed predictors that were present in less than 0.02% of the combined case and control population.^2^ The second step was to use backwards-stepwise logistic regression on the four samples (split by gender and age (15–18, 19–24)) to down select from the remaining predictors based on significance levels (*p* values ≤ 0.01).

## Analysis

In the analysis performed by NRCBM, a backward stepwise logistic regression (using STATA, version 12) was fitted to the training set for each group (using 67% of data in total). The main reported results include odds ratios for each predictor in the analysis, and AUC-ROC curves, which were obtained by cross-validating each model against the remaining 33% of the data.^3^

### Replication

#### Data

New dataset was obtained from THIN for the purpose of this replication project. Our data included records gathered between 1st of December 2008 and 30th November 2020. Notably, although we do not know the details of the data request/purchase originally made by NRCBM, we are certain that our own request was different. More specifically and due to financial constraints, we obtained data on cases pertaining to depression among young people. To the best of our knowledge, NRCBM had access to a much broader data set, which they then reduced by subsetting to the target disorder and population. This matters because some of the exclusion criteria used by NRCBM do not apply in the present study (at least at the stage of data pre-processing). For example, there was no need in our case to exclude individuals with bipolar disorder, psychosis, and hypomania, simply because our data did not include any such cases. Details of our own exclusion process are summarized in Fig. 1, and this should be compared with the equivalent Fig. 1 in NRCBM.

Despite these differences, we attempted to achieve exactly the same exclusion/inclusion criteria as those employed by NRCBM. In our case, this procedure resulted in records from 168,726 cases and 337,452 matched controls (with the case to control ratio of 1:2). We further removed data that overlapped with the period covered by NRCBM’s dataset, which resulted in the final dataset of 107,043 cases and 214,086 controls. Using the same Read code/drug code definitions as those used by NRCBM, we identified the 54 exposure variables to be used as initial predictor candidates in our analysis. The code sets that defined these exposure variable predictors were obtained directly from the paper’s authors as they are not defined within the paper itself.

As per the original study, predictors were removed based on low prevalence (< 0.02%) first leaving 48, before being removed using the backward stepwise regression. The IMD social deprivation data was missing for approximately 1% of the matched case–control data supplied for the replication. Individuals without IMD data were removed from the training/test data prior to our analysis. Additionally, we found a small number of individuals (fewer than 10 in any of the four gender/age group subsets) with an excessive number of visits to the GP. A decision was taken to remove those with a visit count greater than 50 (per year) as this likely reflects some error in data recording. The total number of cases and controls is shown in Table 1, alongside the figures from NRCBM. These data sets were further divided, as by NRCBM, into 67% training for the development of the backwards stepwise replicated models and 33% test subsets for AUC-ROC estimation.

**Table 1:** Total numbers of cases and controls in NRCBM and current study

Subgroup		NRCBM		Replication	
	Age	Case	Control	Case	Control
Male	15–18	4702	14,074	9427	18,852
	19–24	17,526	51,907	31,088	62,185
Female	15–18	11,857	34,315	18,712	38,686
	19–24	33,236	91,839	46,020	92,061

### Alternative models

Extending the work of NRCBM, we tested alternative ML classification models on our data, using predictors left after down-selecting based on 0.02% prevalence to predict depression. We fitted LASSO (least absolute shrinkage selection operator), Random Forest, Gradient Boosting, XGBoost, Rpart, and PRE (prediction rules ensembles). These models were selected as a representative sample of techniques that are commonly used for prediction problems involving large datasets with large numbers of collinear predictors. In all cases, we report AUC-ROC results following the same cross-validation approach as in the main analysis using the logistic regression, specifically measured using the 33% test subset. Table 5 in Appendix 2 summarizes models that were fitted to the data, along with the details of packages that were used to implement them. Code samples will be made available on request, though it should be noted that the data itself cannot be shared due to copyright and ethical constraints.

## Results

### Demographics and predictors

Table 6 in the Appendix 3 summarizes key demographic information of our sample, comparing it directly to the data from NRCBM. Although we observe some minor differences (e.g., larger proportion of males) we note that it is not possible to determine whether these disparities are due to the changes in the EHRs (e.g., how data are recorded in primary care) or whether they represent some more general time trends.

Overall, we were able to reproduce the steps for identifying predictors using Read codes in combination with drug codes provided by NRCBM. One exception is that the Townsend Deprivation Index used by NRCBM is no longer supplied by THIN, as it was replaced with the Index of Multiple Deprivation (IMD). In Appendix 4 we provide some further details about the similarity/differences between the two. A second point of difference is the number of visits to a General Practitioner (GP), the count predictor. NRCBM doubled the counts in the last 6 months (from the index date) for those individuals who were registered with a GP for less than a year. Since our data includes data from prior registrations this step was not necessary in our case.

Table 7 in the Appendix 5 provides information about the prevalence of all predictors in NRCBM and current replication, separately for cases and controls. In NRCBM, removing predictors with less than 0.02% prevalence resulted in the exclusion of sleep (too much of), divorce, unemployment, teenage pregnancy, family history of abuse or neglect, family history of drug misuse, family history of alcohol misuse, and family history of depression. The same variables were removed in the present dataset, with the exception of sleep (too much of) variable as its prevalence increased considerably for cases (from 0.03 to 0.3), despite being consistent for controls (0.01).

### Stability of stepwise models

We now turn to our primary objective and assess the replicability of the main results reported by NRCBM. Following the removal of predictors with low prevalence, a backward stepwise selection (with 0.01 *p* value cut-off) was applied to the four datasets split by age and gender. Table 2 summarizes the predictors that were included/removed for each group in NRCBM and in the present study.

**Table 2:** Predictor variables that were removed/retained following the backward stepwise selection procedure

	Female 15–18		Female 19–24		Male 15–18		Male 19–24	
Predictor	NRCBM	Rep	NRCBM	Rep	NRCBM	Rep	NRCBM	Rep
imd quintile	✓	✓	✓	✓	✓		✓	✓
Abdominal pain	✓		✓				✓	
Alcohol misuse			✓	✓			✓	✓
Anxiety	✔	✔	✔	✔	✔	✔	✔	✔
Asthma			✓	✓				✓
Back pain, with specific symptoms			✓	✓				✓
Back pain without specific symptoms	✓		✓	✓	✓		✓	✓
Bed wetting					✓			
Bereavement	✔	✔	✔	✔	✔	✔	✔	✔
Carer (young)				✓				
Count	✔	✔	✔	✔	✔	✔	✔	✔
Developmental issues							✓	
Diabetes		✓	✓	✓		✓	✓	✓
Drug misuse		✓	✓	✓	✓	✓	✓	✓
Dysmenorrhea			✓		n/a	n/a	n/a	n/a
Dyspepsia	✔	✔	✔	✔	✔	✔	✔	✔
Eating disorders	✓	✓	✓	✓			✓	✓
Emotion (childhood problems)								
Epilepsy							✓	
Excessive sweating			✓					
Headache	✔	✔	✔	✔	✔	✔	✔	✔
Homeless								✓
Ill-defined conditions		✓		✓				
Loss of enjoyment			✓	✓				✓
Low mood	✔	✔	✔	✔	✔	✔	✔	✔
Missing smoker data	✔	✔	✔	✔	✔	✔	✔	✔
Missed immunization								
Neonatal problems			✓				✓	
Non-accidental injury	✓	✓	✓	✓	✓	✓	✓	
OCD	✔	✔	✔	✔	✔	✔		✔
Other somatic symptoms		✓		✓				
Psychosexual problems							✓	✓
PTSD	✓	✓	✓	✓		✓	✓	✓
Puberty (early/late)								
School problems	✓	✓			✓	✓		
Self-harm	✔	✔	✔	✔	✔	✔	✔	✔
Skin problems		✓		✓		✓		✓
Sleep, too little	✔	✔	✔	✔	✔	✔	✔	✔
Sleep, too much		✓		✓				✓
Smoker	✔	✔	✔	✔	✔	✔	✔	✔
Social services involvement				✓	✓			✓
Tiredness	✓	✓	✓	✓	✓		✓	
Weight gain								
Weight loss			✓			✓	✓	✓
Work stress			✓	✓			✓	✓
Total (of 48)	22	26	32	33	22	18	29	31
Common predictors (*n*)	20		27		14		23	
Correlation (phi)	0.66		0.46		0.59		0.39	

Note 1: Dysmenorrhea does not present in males

Note 2: Predictors in bold common across all models in both NRCBM and this replication

Note 3: For interpretation of phi: 0.01 to 0.19—no or negligible, 0.20 to 0.29—weak, 0.30 to 0.39—moderate, 0.40 to 0.69—strong 0.70 or higher-very strong positive relationship [58]

Overall, the backward stepwise selection procedure produces similar results between the predictors used in the present study and those used by NRCBM. Indeed, the correlation (phi) between the NRCBM and replication predictors for F 15–18, F 19–24 and M 15–18 indicates a strong positive relationship, and a moderate relationship for the M 19–24 group. Despite these correlations, there are some notable differences between selected predictors. In fact, many seemingly relevant predictors are not consistently identified. For example, drug misuse in the F 15–18 group was retained as a predictor in the present study but did not survive the stepwise procedure in the NRCBM analysis. Conversely, tiredness featured as a predictor in the final model for the M 15–18 group, but this variable was excluded in the present study. Some other relevant variables that are not consistent in this manner include weight loss, PTSD, and other somatic symptoms.

**Fig. 2:**
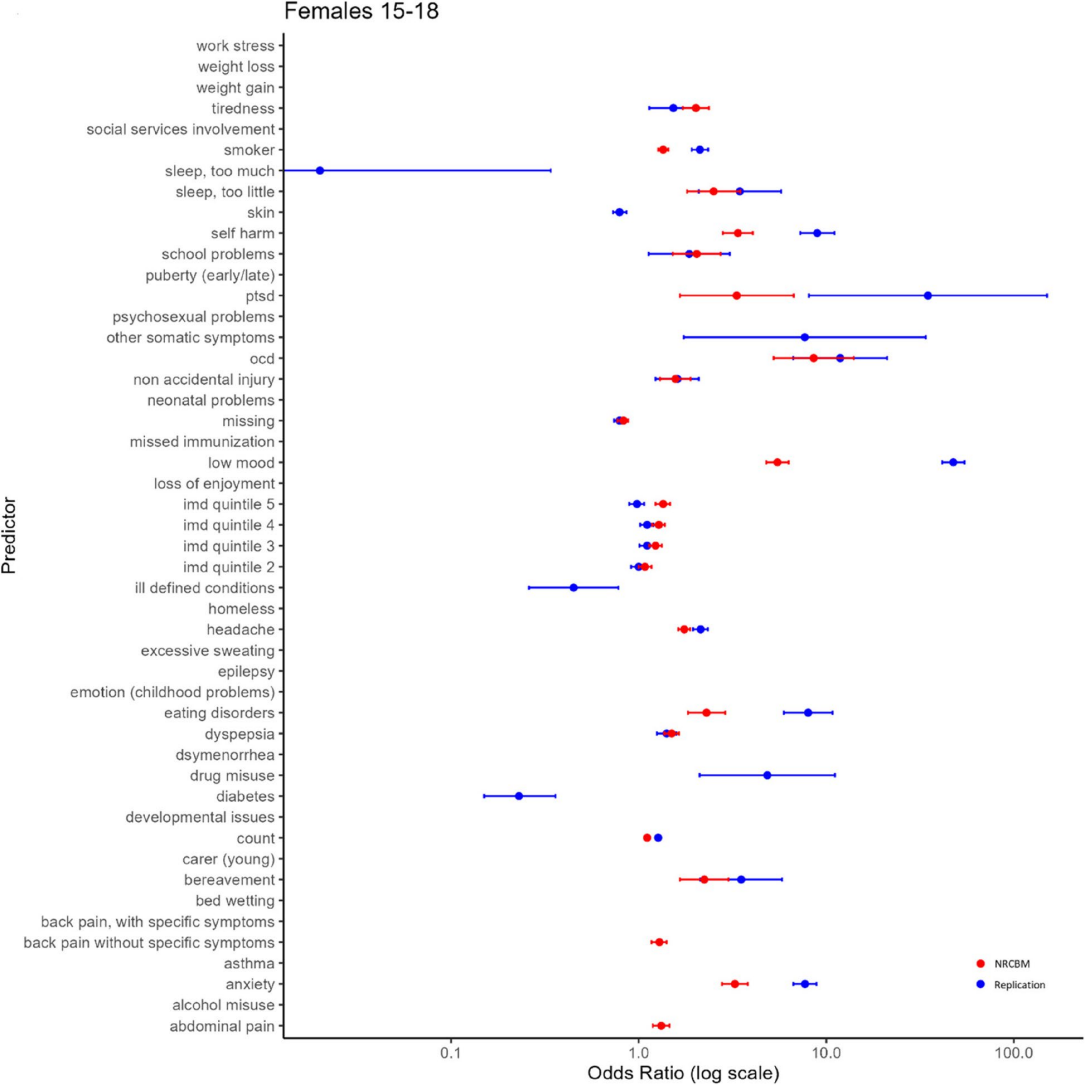
Odds ratio vs predictor for females ages 15–18

**Fig. 3:**
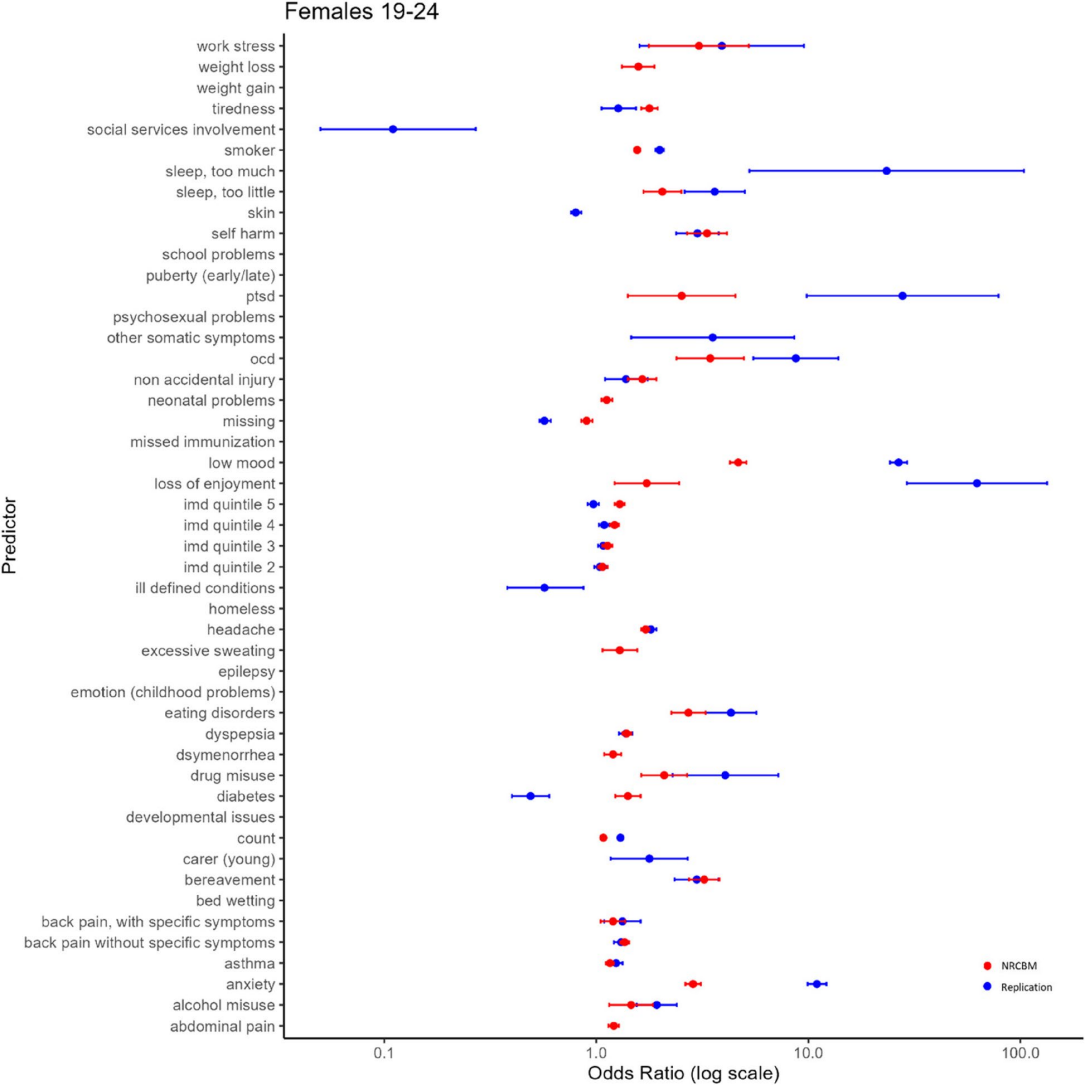
Odds ratio vs predictor for females ages 19–24

**Fig. 4:**
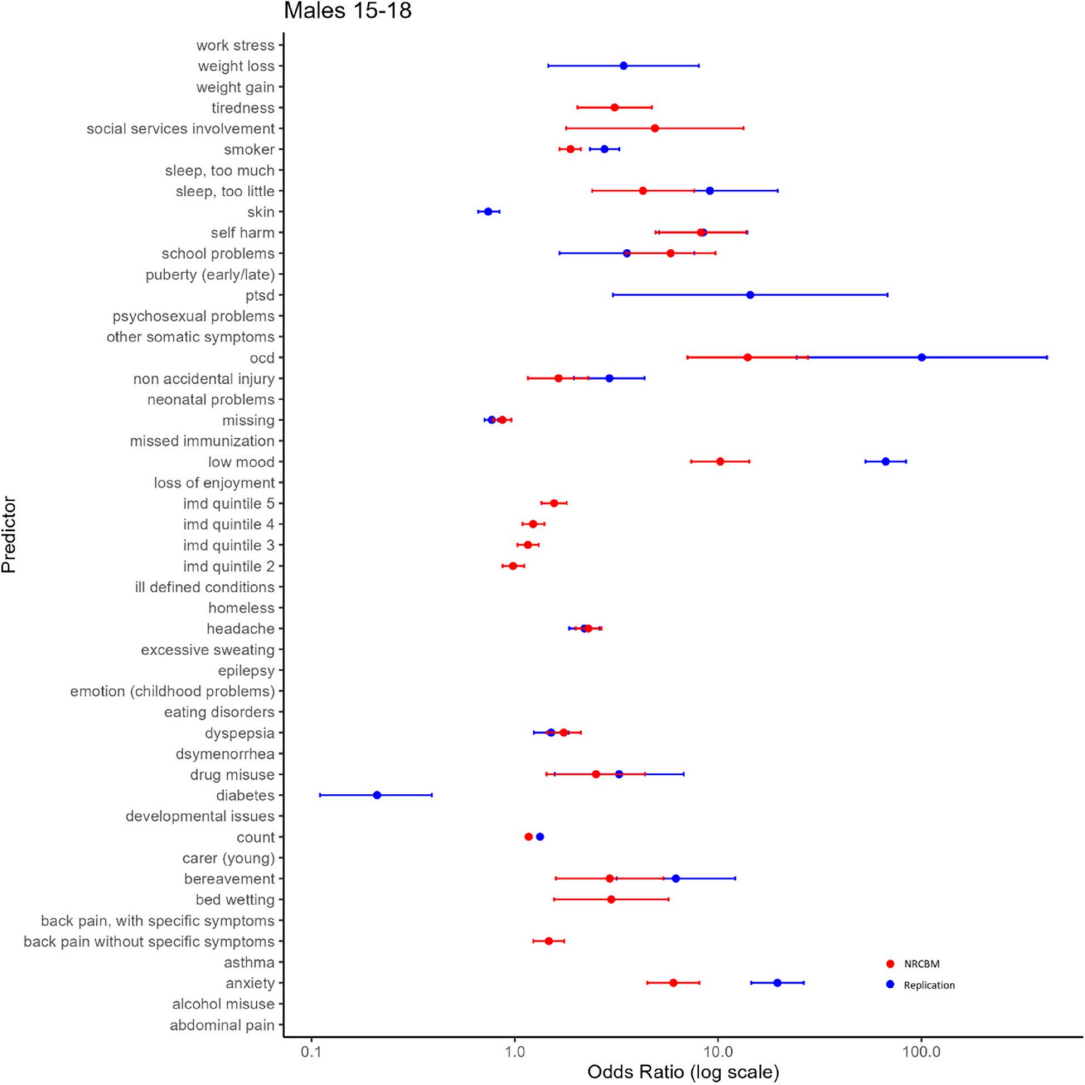
Odds ratio vs predictor for males ages 15–18

**Fig. 5:**
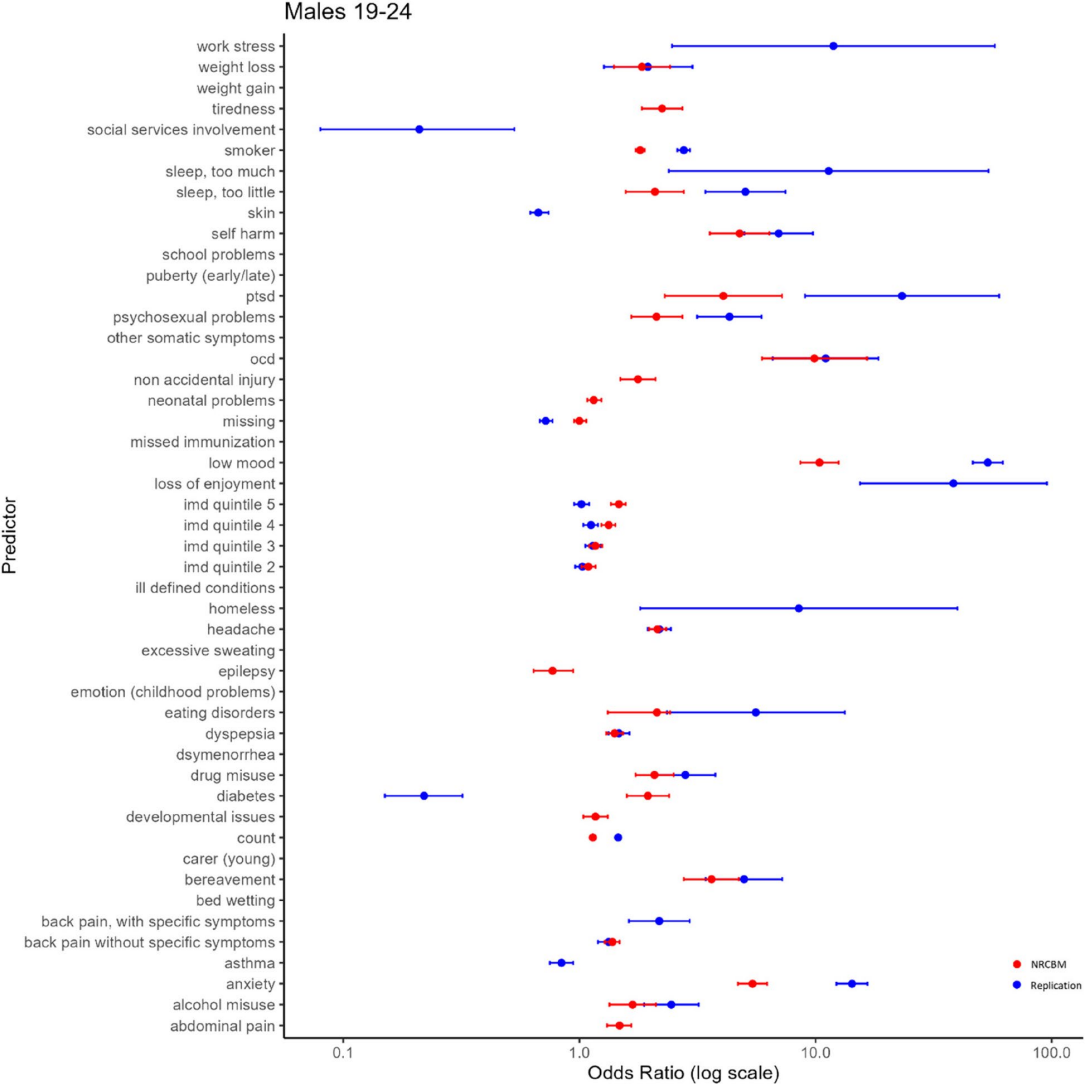
Odds ratio vs predictor for males ages 19–24

We now turn to the modelling results and compare the odds ratios (ORs) for each predictor used in the replication and in the NRCBM’s study, separately for the four demographic groups. These results are summarized in Figs. 2, 3, 4, and 5.

Overall, the pattern of ORs is qualitatively similar between NCRBM and the replication data set. Predictors with a relatively large OR in NCBRM generally also received a relatively large OR in the replication. Indeed, a rank correlation between ORs is relatively high, with the Spearman’s rho ranging from 0.82 to 0.93. As expected and consistent with the results of NRCBM, anxiety, self-harm, loss of enjoyment, PTSD, OCD, and low mood are among largest predictors of depression.

However, there are important quantitative differences with the replication data set in many cases producing much larger ORs. For example, the low mood predictor appears with a much higher ORs in the replication than in the original study. According to our results, a record of experiencing depression makes the odds of a young person having depression over 40 times higher than a control if they are identified with low mood. The ORs obtained in the present study are also substantially higher for eating disorders. For example, in the case of younger females (F 19–24), the OR of 8.91 indicates that patients with a diagnosis of eating disorders have 8.91 odds of being diagnosed with depression than a control. This is considerably higher than the results of NRCBM, where the odds are 2.31. Across all predictors, the differences are substantial. The mean sizes of the coefficients in NRCBM are considerably lower than those of the replication (F 15–18 NRCBM 2.42, Rep. 7.10; F 19–24 NRCBM 1.97, Rep. 6.62; M 15–18 NRCBM 4.45, Rep. 16.36; M 19–24 NRCBM 2.87, Rep. 6.56). In summary, although the relative importance of the variables is similar between the datasets, magnitude differences of individual coefficients are large.

We now turn to the overall predictive accuracy of the estimated models. From the comparison of the coefficients, one could expect that as long as the models pick up on the relevant variables, then the models should be able to predict depression accurately. The differences in magnitudes may therefore simply reflect changes in prevalence that occurred between the periods covered by the two datasets or other idiosyncratic aspects of the data. Indeed, AUC-ROC curves reveal that the replication models performed very well with the AUCs of 0.886 (F 15–18), 0.880 (F 19–24), 0.882 (M 15–18), and 0.887 (M 19–24). Interestingly, these performances are higher than those obtained by NRCBM, who reported AUCs of 0.719 (F 15–18), 0.699 (F 19–24), 0.714 (M 15–18), and 0.716 (M 19–24). We return to these somewhat surprising results in the “General discussion” section.

Following analysis of NRCBM, we have also performed a sensitivity analysis, by removing depression specific predictors (anxiety, bereavement, low mood, self-harm, OCD and PTSD) and re-estimating our models. In summary, we found a small reduction of AUCs across the board (F 15–18 = 0.067, F 19–24 = 0.039, M 15–18 = 0.067, M 19–24 = 0.038), which appears in line with NRCBM, who claimed that the analysis “resulted in only minor differences in the variables included and estimates of effect.”

Finally, we evaluated out of sample prediction of the models estimated by NRCBM on our own data. Using their estimated coefficients to make predictions for each group in our data, we find very high AUC scores 0.870 (F 15–18), 0.860 (F 19–24), 0.845 (M 15–18), and 0.847 (M 19–24). It therefore appears that the model trained by NRCBM performs better on the newer dataset than it did on the slice of the test data from the same period when the model was trained. This result further suggests that the exact magnitudes of the ORs are indeed not as relevant as long as the relevant predictors are selected, and the magnitudes are in the correct ballpark.

### Alternative models

We tested six alternatives to the backward stepwise regression on our data: Least Absolute Shrinkage Selection Operator (LASSO), Random Forrest (RF), Gradient Boosting (GB), XGBoost (XGB), Rpart, and Prediction Rules Ensembles (PRE). Models were trained and evaluated in exactly the same way as the logistic regressions reported earlier, obtaining AUCs scores via cross-validation. Table 3 summarizes these results for each group.

**Table 3:** Alternate model performance on replication data test set for M/F by 15*–*18/19*–*24 subsets

Model set	LASSO	RF	GB	XGB	Rpart	PRE	Stepwise replication
F 15–18	0.887	0.882	**0.891**	0.885	0.838	0.890	0.886
F 19–24	0.880	0.880	**0.887**	**0.887**	0.848	0.886	0.880
M 15–18	0.881	0.881	**0.889**	0.886	0.863	0.888	0.882
M 19–24	0.895	0.895	0.898	**0.902**	0.873	0.898	0.887

*Note*: The highest AUC values are highlighted in bold font

Overall, we find the XGBoost performs best on two out of four datasets, Gradient Boosting best on three, with one tied result. However, the overall performance is comparable across all models. Indeed, the difference between the best-performing and the stepwise logistic regression (Stepwise Replication column) is rather small, ranging between 0.005 and 0.015. In short, despite using much more advanced and recent regression frameworks, little additional value was found over the predictive accuracy obtained with a logistic regression model.

### GP visits

Although our alternative models achieved comparable performance to the standard logistic regression, it is an open question whether these models agree with respect to the variable importance. Using “varimp” function in R (using the caret library (version 6.0–92)), we computed relative importance for all of models discussed so far. We report the full ranking obtained for each model and each group in Appendix 6, but we use the remainder of this section to discuss the “count” variable, which corresponds to the number of visits to GP within a year prior to the index date. Our results show that count consistently appears as one of the highest-ranking predictors in terms of variable importance (Tables 8 and 9, Appendix 6). In fact, removing count from the list of predictors in our models largely reduces the AUC performance. To illustrate, consider some of the models fitted to the F 15–18 group. Here we found that our AUC reduces from 0.886 to 0.828 for the backward stepwise logistic regression, from 0.838 to 0.786 for Rpart, and from 0.890 to 0.828 for Pre. Thus overall, the count variable seems to have a non-trivial effect on model accuracy. We discuss the reason why count may be so important in the final section.

## General discussion

In recent years, the growing popularity of ML methods and the expanding accessibility of large healthcare datasets resulted in many new efforts to train predictive models of mental and physical health diagnoses. Yet, little or no effort has been made to reproduce and replicate existing research. To address this issue, the goal of the present paper was to establish the robustness of findings showing that EHRs can be used to accurately predict diagnoses of depression among young adults. To this end, we obtained a large dataset of EHRs and trained new regression models following the methodology of Nichols et al. [48], (referred to as NRCBM). With some minor exceptions (which we elaborate on further on), we were able to perform the same analyses as NRCBM on a newer set of EHRs. Regardless of whether we fitted new models to our data, or whether we applied NRCBM’s models to make out-of-sample predictions, we found that the models’ accuracy was both high and comparable with the previous work. In addition, we were able to test the robustness of the original result by fitting alternative models to the data. While our efforts were successful, a number of issues and challenges emerged during our analysis. These issues are largely unspecific to the work of NRCBM but rather reflect the general challenge posed by working with big healthcare data and ML tools. We discuss each challenge in turn.

Our replication was possible because we were able to access a large set of EHRs, containing data from over 350,000 individuals. These data, like the dataset obtained by NRCBM, are largely representative of the UK population, which means that the results of modeling are suitable for concluding UK population. In addition, we could avoid any overlap with the dataset used in the work we sought to replicate, which allowed us to perform appropriate out-of-sample tests. Yet, it has to be noted that the availability of similar datasets for other researchers can be highly restrictive. The scope of the current dataset was constrained by the research budget (£17,000), which for example, limited us to a smaller number of controls available for each case. Although we were fortunate to have sufficient funds to replicate the existing work of NRBCM, we were not able to obtain the data necessary to replicate the results of NRCBM on the *same* EHRs. Additionally, the restrictions on intellectual property and confidentiality mean that these data cannot be shared with other researchers. We chose to obtain newer data largely due to the value of being able to make out-of-sample predictions. Nonetheless, we note that most researchers (us included) would not have the resources to replicate other similar work simply due to resource limitations.

Whereas we were able to obtain necessary EHRs from the THIN’s database, we did not have access to the NHS Read code/drug that is necessary to define each predictor. Fortunately, we were able to obtain these from the authors directly. If we were not able to access these, we would have much less confidence that our analysis and results match those reported by NRCBM. It is therefore essential precise definitions of each predictor are included in other work that relies on EHRs.

The key factor that can affect the replicability of large data projects is data exclusion. Following NRCBM, the first step in data preparation required the removal of predictors with low (< 0.02%) prevalence. Unlike NRCBM, we found that “sleep (too much)” had to be retained for analysis because of a tenfold increase in its prevalence (from 0.03 to 0.3%). Still, considering that this represents merely a few hundred individuals, we conclude that the stability of predictor prevalence is relatively high. The second step of the analysis involved the removal of predictors on the basis of the backwards stepwise logistic regression. While we found an overall similar pattern (the lowest correlation between selected predictors in NRCBM and our dataset was 0.39, and the highest was 0.66), we also found important disparities from the original research. These results support a widespread criticism of the stepwise procedures, especially in the context of large datasets with large sets of predictors [59]. With the popularity of regularized regression frameworks, it seems that there is little value in relying on arbitrary thresholds for deciding which predictors to retain and which to remove.

Robustness of the original findings was determined using two methods. First, NRCBM reported an average AUC across four groups of 0.712 (SD = 0.009). For the replication, the average AUC for the regression using the backwards-stepwise selected predictors was 0.88 (SD 0.004). This is a considerable and surprising improvement in model accuracy. Even more surprising is the fact that applying the model estimated by NRCBM to our data also leads to an improvement in the average AUC (0.855, SD = 0.012). One possibility is that these results reflect the increased use of “low mood” in EHRs. Indeed, the prevalence of low mood was higher among cases in our dataset relative to the NRCBM’s sample (5.90% in the NRCBM “case” dataset vs 25.73% in the new case dataset, the respective values in the control datasets were 0.84% and 0.77% respectively). There is some evidence that the use of low mood in EHRs changed over the years. First, there were changes to the contract for NHS primary care providers in 2006/7 [60]; treatment of depression changed significantly in response to the Quality Outcome Framework (QOF), and guidelines were further updated in 2009 [61], just ahead of the start date in the replication dataset. How could these changes influence the EHRs? One possibility is that, in some cases, recording of low mood by primary care practitioners could have replaced initial diagnoses of depression. Consistent with this view, depression prevalence decreased shortly following the 2008 economic recession, which aligns with the time when practitioners were more likely to use low mood as an initial diagnosis (in response to the 2006 QOF update [62]). Although largely speculative, there are two reasons why low mood could have replaced diagnoses of depression following the QOF update. First, although the QOF change was initially well received by practitioners, the update put forward strict requirements on the timing for screening and severity assessments following a depression diagnoses. At the risk of missing these targets, practitioners could be more motivated to rely on the low mood diagnosis instead [63]. Second, and in a similar vein, a perceived lack of resources for providing necessary cognitive behavioral therapy could further encourage practitioners to opt for the low mood diagnosis [64].

Despite their popularity, applications of ML methods to clinical data have been criticized for several reasons. One important issue with many ML models is that they can be unstable with respect to the variable selection, weights associated with each predictor, or the models’ performance (both on the aggregate and individual level) [65]. Although there are many reasons why models could be unreliable, a common issue is insufficient amount of data—a problem that is particularly relevant for the modern classification methods [66]. Nonetheless, we doubt that that this issue applies to the present work (or to the work of NRCBM) on account of the very large sample sizes used (minimum group size of 28,279). These sample sizes are large enough to minimize the error that can occur if either the number of observations (cases and controls) is low, or due to a sparse number of events per predictor (e.g., very low number of people with anxiety or diabetes). In addition, it is notable that the results in the present paper are not only consistent with the LRs reported by NRCBM, but are also consistent across many diverse ML techniques that were used. Still, future work should consider both the replicability and stability of ML applications in clinical settings.

One limitation of the present (and previous) study is the operationalization of depression. To select the cases from the THIN database, NRCBM used a combination of diagnostic NHS Read codes or prescription of antidepressant drugs. This is a widely used method, see e.g., [67], but it may be too wide in scope; antidepressant drugs are also prescribed for other disorders including those with chronic pain [68, 69], OCD [70, 71], PTSD [72, 73] and anxiety [74, 75]. The reported range for off-label prescribing of antidepressants, where an antidepressant drug is prescribed for non-licensed purposes, is 25 to 35% [76, 77], though figures of over 88% have also been reported [73]. Sarginson et al. [78] identified that for 15- to 17-year-olds females, there had been a rapid increase in first-time antidepressant prescriptions for both depression and non-depression-related conditions between 2000 and 2015, further indicating that some cases in the original and replication datasets may not accurately reflect depression. Using a definition of depression that is too broad may harm the out-of-sample accuracy of the model. This is a significant limiting factor and an important consideration for future ML diagnostic/prediction applications using EHRs.

Our consideration of the count variable shows that researchers should think carefully about whether to include information about GP visits in their models. As expected, visits to the GP are more prevalent among cases than controls (5.07 vs. 1.53 per annum on average). However, there is no reason to believe that these visits are uniquely related to depression or even mental health in general. Indeed, in the UK, 10% of patients are responsible for 40% of primary care visits across multiple disorders [79]. A cursory look at the existing literature shows that GP visits are often used in predictive models of depression [67, 80, 81]. Although this may be warranted for many research questions, researchers should be careful in interpreting the importance of the count information. The inclusion of this variable may be more justified if the researchers wish to maximize the predictive power of their model, not when their goal is to understand the unique psychobiological precursors of depression (or build a model that can predict more than one disorder). Including a general variable indicating any severe health issues such as count may also be more helpful in a differential prediction model that does not only try to predict one disease against control but also tries to make a differential prediction among multiple diseases.

Despite fitting multiple models that improve on the standard backward stepwise regression, the alternative models showed no major improvement in predictive accuracy. This result is in line with the findings of Christodoulou et al. [82] who reviewed over 70 studies where ML was used to predict a binary outcome and found no significant benefit in terms of AUC predictive performance vs logistic regression. Overall, our results suggest that a simple logistic regression can suffice in the context of EHRs. Our results in out-of-sample prediction are also encouraging, as they indicate some stability of predictive models across time.

## Conclusion

Although we demonstrated that the variable selection is not exactly stable when using a backwards stepwise logistic regression, overall, our results aligned well with the original study. This was the case both for the replication of the original model and the out-of-sample replication applying NRCBM coefficients to our new EHRs data. We believe we are the first to carefully replicate ML analysis on EHRs to predict depression among young people. In replicating and extending the depression prediction models of NRCBM we have contributed to the debate about the suitability of using EHRs to inform the development of early diagnosis for adolescents and young adults. We showed that stepwise logistic regression performs comparably well to more advanced types of (regularized) regressions and ensemble methods. Through our analysis, we demonstrated some potential issues associated with the reliance on EHRs, including changes in the regulations and guidelines (such as the QOF guidelines in the UK) and reliance on visits to GP as a predictor of specific disorders. These issues are illustrative of the challenges faced by researchers who may be interested in predicting health diagnoses using large datasets of primary health records.

## Data Availability

The data that support the findings of this study are available from THIN (50) but restrictions apply to the availability of these data, which were used under license for the current study, and so are not publicly available. The definitions used to obtain the data are available from the authors on request and, subject to commercial and ethical constraints may be available from THIN (50). Code vignettes will be made available  via this Open Science Framework link https://osf.io/573uw/.

## Appendix 1

### Predictors (exposure variables)

**Table 5:** Summary of all predictors used by NRCBM categorised into predictor groups

Predictor group	Time	Type	Derivation	Predictors
Any time	Any time prior to index date	Categorical	NHS Read code from disorder-related list	Developmental delay, early childhood emotional problems, missed immunizations, neonatal health problems, and early/late puberty
Two year	Within two years prior to index date	Categorical	NHS Read code and/or disorder drug code list	Anxiety, low mood, tiredness, loss of enjoyment, too little sleep, too much sleep, eating disorders, weight loss, weight gain, bed wetting, excessive sweating, self-harm, headache, dyspepsia, dysmenorrhea, abdominal pain, back pain, ill-defined conditions, other somatic symptoms, skin problems, divorce, homelessness, bereavement, unemployment, family history of abuse or neglect, family history of drug misuse, family history of alcohol misuse, family history of depression, abuse/neglect/non-accidental injury, police involvement, other social services involvement, psychosexual problems, school problems, teenage pregnancy, work stress and young carer. Asthma, diabetes, dyspepsia and epilepsy
Smoker, missing	Within two years prior to index date	Categorical	NHS Read code (covers smoker, ex-smoker and never smoked), or “Missing” meaning no data	Smoker Missing
Count	Within one year prior to index date	Integer	Match person to GP contact database to obtain the number of visits in the year	Count
Townsend Index of Deprivation	At time of data extraction	Quintile (Integer 1 to 5)	Match person to practice Identifying quintile	Deprivation Index (IMD)

Note: For details on NHS Read codes see SCIMP [57]

## Appendix 2

### Alternative ML models

**Table 5:** Alternative ML models, notes, and implementation library

ML Model	Notes	Implementation library for R (v 4.1.3)
LASSO (Least Absolute Shrinkage Selection Operator)	A regression model but, unlike stepwise logistic regression in the NRCBM study, it uses a regularization term to penalize complex models thus supporting the selection of only the more important predictors	library (glmnet) 4.1–3
Random Forest	An ensemble learning method supporting regression and classification. Creates multiple decision trees based on subsets of training data, then uses them to make predictions based on mode/mean of individual trees	library (randomForest) 4.7–1
Gradient Boosting	An ensemble approach that combines predictions form multiple weaker models such as decision trees or regression models, using a gradient descent method to improve accuracy. It is suitable for both classification and regression applications	library (gbm) 2.1.8
XGBoost	This is another ensemble approach but, unlike Gradient Boosting it uses a Newton–Raphson function and special penalization techniques for tree selection. It is considered to offer improved performance vs e.g., Gradient Boosting, but at the expense of interpretability	library (xgboost) 1.5.2.1
Rpart	“Recursive partitioning” is a decision tree algorithm for generating classification, regression and survival trees. The resulting decision trees are considered easy to interpret	library (rpart)4.1.16
PRE (Prediction Rules Ensembles)	Used for both regression and classification, models are based on a combination of very simple, “if x then predict y” rules. The aim of PRE is to aim to optimize both accuracy and interpretability	library (pre)1.0.4
Stepwise Logistic Regression (original model)	Stepwise regression model based on the logit function used with pre-specified predictors for classification	library (rms) 6.6–0

Note: Fuller descriptions of these methods are available in the documentation accompanying the libraries and in other sources such as ML papers and textbooks. Code vignettes  will be made available via this Open Science Framework link: https://osf.io/573uw/

## Appendix 3

### Demographic comparison

**Table 6:** Demographics, Ethnicity, Sex, Age, Social deprivation

	NRCBM Study %	Replication Study %
Ethnicity		
White	32.77	39.73
Black	1.00	1.50
Asian	1.82	2.29
Mixed	0.51	0.85
Chinese	0.24	0.40
Other	0.51	0.85
Missing	63.2	54.4
Sex/age		
Male	32.9	36.9
Female	67.1	63.1
Under 19	37.3	25.9
19 and over	62.7	74.1
Deprivation index	Townsend quintile	IMD
1	16.8	15.5
2	15.9	17.2
3	20	17
4	23.6	25.2
5	20	24.1

## Appendix 4. Townsend vs IMD deprivation indices

For the Townsend Index individual areas are assessed against a set of four deprivation criteria, for example unemployment, car ownership, and home ownership, then given a ranking of 1 to 5. For the IMD the individual areas are ranked for a larger number of similar, but not identical, criteria continuously across the country from least to most; this can then be subdivided into quintiles to give the equivalent of the Townsend 1 to 5 index value. IMD is a more sophisticated measure but has been shown to be broadly equivalent and is thus considered an acceptable replacement (Chapter 8: A comparison of deprivation indices: Townsend 4 and Index of Multiple Deprivation 2004. (n.d.)).

## Appendix 5

### Predictor prevalence comparison

**Table 7:** Predictor prevalence in NRCBM and the non-overlapping replication datasets

Predictor	NRCBM case %	NRCBM control %	Replication case %	Replication control %	Case difference	Control difference
Anxiety	4.99	1.10	9.24	0.76	4.25	− 0.34
Asthma	15.87	10.66	8.37	3.42	− 7.50	− 7.24
Back pain with specific characteristics	1.38	0.65	1.07	0.30	− 0.31	− 0.35
Back pain without specific characteristics	11.00	5.75	8.37	2.86	− 2.64	− 2.89
Bed wetting	0.12	0.07	0.09	0.08	− 0.03	0.01
Bereavement	1.19	0.30	0.91	0.17	− 0.28	− 0.14
Developmental delay	2.29	1.92	2.77	2.24	0.49	0.32
Diabetes	0.13	0.61	0.60	0.74	0.47	0.12
Dysmenorrhea	3.11	2.02	2.07	0.95	− 1.04	− 1.07
Dyspepsia	12.16	5.37	9.94	3.01	− 2.22	− 2.36
Eating disorders	0.92	0.25	1.01	0.12	0.09	− 0.13
Emotion	0.06	0.02	0.06	0.03	0.00	0.01
Epilepsy	1.25	0.83	1.33	0.34	0.07	− 0.50
Headache	12.16	5.37	12.7	3.79	0.54	− 1.58
Homeless	0.08	0.03	0.07	0.00	− 0.01	− 0.03
Alcohol misuse	0.76	0.31	1.15	0.25	0.38	− 0.06
Drug misuse	1.01	0.29	0.72	0.09	− 0.29	− 0.20
Loss of enjoyment	0.13	0.05	1.19	0.01	1.05	− 0.04
Low mood	5.90	0.84	25.73	0.77	19.83	− 0.07
Missed immunization	0.67	0.57	2.26	1.85	1.59	1.28
Neonatal	8.77	7.62	10.38	8.46	1.61	0.84
Non-accidental injuries	1.86	0.73	1.19	0.45	− 0.66	− 0.28
OCD	0.44	0.07	0.56	0.04	0.11	-0.03
Social Services Involvement	0.08	0.06	0.20	0.14	0.12	0.08
Police involvement	0.05	0.02	0.05	0.01	0.00	− 0.01
Psychosexual problems	0.30	0.11	0.31	0.06	0.01	− 0.05
PTSD	0.19	0.04	0.34	0.01	0.15	− 0.03
School problems	0.34	0.10	0.24	0.06	− 0.10	− 0.04
Self-harm	1.50	0.29	2.35	0.26	0.85	− 0.03
Skin disorders	14.49	11.8	12.8	7.84	− 1.69	− 3.96
Sleep disorder, too little	0.90	0.22	1.20	0.09	0.31	− 0.13
Sleep disorder, too much	0.03	0.01	0.30	0.01	0.27	0.00
Teenage pregnancy	0.02	0.01	0.00	0.00	− 0.01	− 0.01
Tiredness	2.94	1.16	1.19	0.37	− 1.76	− 0.80
Unemployment	0.01	0.00	0.01	0.00	0.00	0.00
Other somatic symptoms	0.11	0.03	0.10	0.01	− 0.01	− 0.02
Ill-defined conditions	0.51	0.28	0.23	0.11	− 0.27	0.17
Weight gain	0.18	0.10	0.08	0.03	− 0.10	− 0.07
Weight loss	0.90	0.35	0.59	0.13	− 0.30	− 0.23
Work stress	0.08	0.02	0.06	0.01	− 0.02	− 0.01
Carer	0.08	0.06	0.23	0.07	0.15	0.01
Smoker	38.12	24.2	23.9	7.04	− 14.22	− 17.17
Missing (smoker data)	15.29	22.93	12.39	26.40	− 2.90	3.47
Divorce	0.01	0.00	0.00	0.00	− 0.01	0.00
Abdominal pain	7.61	3.7	4.92	1.64	− 2.69	− 2.07
Excessive sweating	0.59	0.37	0.58	0.23	− 0.01	− 0.14
Puberty, early or late	0.23	0.19	0.34	0.26	0.11	0.06
Family history of depression	0.00	0.00	0.04	0.00	0.04	0.00
Family history of drug abuse	0.01	0.00	0.00	0.00	− 0.01	0.00
Family history of alcohol abuse	0.00	0.00	0.00	0.00	0.00	0.00
Family History of abuse	0.01	0.00	0.03	0.01	0.02	0.01

Note: The visit “count” predictor is not included as it is, by definition, 100% in all groups

## Appendix 6

### Count ranking in different models

**Table 8:** Rpart, Prediction Rules Ensemble, and logistic regression model variable importance rankings

Model/predictor	Rpart variable importance rank	Prediction rules ensemble variable importance rank	Replication logistic regression variable importance rank
Anxiety	1	3	3
Count	2	2	2
Eating disorder	3	7	7
Headache	4	6	5
Low mood	5	1	1

**Table 9:** Count comparison ranked predictors by odds ratio–F 15–18 regression models

	Odds ratios			Ranked		
Model Predictor	NRCBM Stepwise logistic regression	Replication logistic regression	LASSO	NRCBM Stepwise logistic regression	Replication logistic regression	LASSO
OCD	8.57	17.1	10.29	1	2	2
Low mood	5.49	45.21	45.90	2	1	1
Self-harm	3.38	10.07	8.58	3	4	4
PTSD	3.33	16.54	9.53	4	3	3
Anxiety	3.26	8.60	7.19	5	5	5
Sleep disorder (too little)	2.51	3.88	4.61	6	7	8
Eating disorder	2.30	7.67	4.29	7	6	9
Bereavement	2.24	3.06	2.82	8	9	12
School problems	2.04	3.34	2.07	9	8	16
Tiredness	2.02	1.54	1.32	10	12	24
**Count**	**1.11**	**1.26**	**1.26**	**20**	**15**	**26**

Note 1: the count (GP visit frequency) predictor is highlighted in bold

Note 2: LASSO did not eliminate as many predictors as did the Stepwise models

## Abbreviations

AUC-ROC: Area under the curve-receiver operating characteristic
BSREC: University of Warwick’s Biomedical and Scientific Research Ethics Committee
DALYs: Disability-adjusted life years
EHRs: Electronic Health Records
EPSRC: Engineering and Physical Sciences Research Council
GP: General Practitioner
IMD: Index of Multiple Deprivation
LASSO: Least Absolute Shrinkage Selection Operator
NHS: National Health Service
NRCBM: Nichol’s et al. [48] (Nichols, Ryan, Connor, Birchwood, Marshall)
OCD: Obsessive Compulsive Disorder
OP: Observation period
ORs: Odds ratios
PRE: Prediction Rules Ensembles
PTSD: Post Traumatic Stress Disorder
THIN: The Health Improvement Network
YLDs: Years of healthy life lost due to disability

## Footnotes

1 This pragmatic reason matters for claims about reproducibility and replicability, and we return to it in the general discussion section.

2 NRCBM also investigated trends in univariable odds ratios for each year. We did not use the same method because a) NRCBM did not provide any precise threshold for predictor exclusion using this method, and b) did not in fact exclude any predictors based on this approach.

3 NRCBM also report sensitivity analysis in which they refitted their models after removing specific predictors that are typically associated with depression (anxiety, bereavement, low mood, self-harm, OCD and PTSD).
